# Poliovirus antibodies following two rounds of campaigns with a type 2 novel oral poliovirus vaccine in Liberia: a clustered, population-based seroprevalence survey

**DOI:** 10.1016/S2214-109X(23)00116-X

**Published:** 2023-05-16

**Authors:** Stephen B Kennedy, Grace R Macklin, Gloria Mason Ross, Rocio Lopez Cavestany, Richelot A Moukom, Kathryn A V Jones, Bernardo A Mainou, Moses B F Massaquoi, Mark W S Kieh, Ondrej Mach

**Affiliations:** a University of Liberia-Pacific Institute for Research & Evaluation (UL-PIRE) Africa Center, University of Liberia, Monrovia, Liberia; b West African Consortium for Clinical Research on Epidemic Pathogens (WAC-CREP), Monrovia, Liberia; c Polio Eradication, World Health Organization, Geneva, Switzerland; d World Health Organisation African Regional Office, Brazzaville, Republic of the Congo; e US Centers for Disease Control and Prevention, Atlanta, USA

## Abstract

**Background:**

Novel oral poliovirus vaccine type 2 (nOPV2) was administered in Liberia in response to an outbreak of circulating vaccine-derived poliovirus type 2 (cVDPV2) in 2021. We conducted a serological survey of polio antibodies after two national campaigns with nOPV2.

**Methods:**

This clustered, cross-sectional, population-based seroprevalence survey was conducted in children aged 0–59 months, more than 4 weeks after the second nOPV2 vaccination round. We used a clustered sampling method in four geographical regions of Liberia, followed by a simple random sampling of households. One eligible child was randomly selected per household. Dried blood spot specimens were taken and vaccination history was recorded. The antibody titres against all three poliovirus serotypes were assessed using standard microneutralisation assays done at the US Centers for Disease Control and Prevention in Atlanta, GA, USA.

**Findings:**

Analysable data were obtained from 436 (87%) of 500 enrolled participants. Of these, 371 (85%) children were reported via parental recall to have received two nOPV2 doses, 43 (10%) received one dose, and 22 (5%) received no doses. The seroprevalence against type 2 poliovirus was 38·3% (95% CI 33·7–43·0; 167 of 436 participants). No significant difference was observed between type 2 seroprevalence in children aged 6 months or older who were reported to have received two doses of nOPV2 (42·1%, 95% CI 36·8–47·5; 144 of 342), one dose (28·0%, 12·1–49·4; seven of 25), or no doses (37·5%, 8·5–75·5; three of eight; p=0·39). The seroprevalence against type 1 was 59·6% (54·9–64·3; 260 of 436), and the seroprevalence against type 3 was 53·0% (48·2–57·7; 231 of 436).

**Interpretation:**

Unexpectedly, the data showed low type 2 seroprevalence after two reported doses of nOPV2. This finding is probably affected by the lower oral poliovirus vaccine immunogenicity previously demonstrated in resource-limited settings, with high prevalence of chronic intestinal infections in children and other factors discussed herein. Our results provide the first assessment of nOPV2 performance in outbreak response in the African region.

**Funding:**

WHO and Rotary International.

## Introduction

The WHO African region was declared free from wild poliovirus in August, 2020.^1^ However, the continued use of the live, attenuated Sabin oral poliovirus vaccine has led to outbreaks of circulating vaccine-derived poliovirus (cVDPV) strains.^2^, ^3^ In 2021, there were 682 paralytic poliomyelitis cases of type 2 cVDPV (cVDPV2) reported across three WHO regions—80% of these in the African region.^4^, ^5^ Accordingly, the spread of cVDPV2, alongside wild poliovirus, remains a Public Health Emergency of International Concern.^6^

Since wild poliovirus type 2 was declared eradicated in 2015, the use of the type 2 component of Sabin oral poliovirus vaccine in routine immunisation has ceased. A global switch from trivalent oral poliovirus vaccine (tOPV, containing types 1, 2, and 3) to bivalent oral poliovirus vaccine (bOPV, containing types 1 and 3) was conducted in May, 2016.^7^ As a risk mitigation strategy, at least one dose of inactivated poliovirus vaccine (IPV) was introduced in all routine schedules to protect against paralysis from type 2 poliovirus. As IPV provides limited intestinal mucosal immunity necessary to stop transmission, a type 2 oral poliovirus vaccine is needed for outbreak response to cVDPV2.^7^, ^8^ Since 2016, the use of Sabin monovalent oral poliovirus vaccine type 2 (mOPV2) in outbreak response has generated an increasing number of cVDPV2 emergences with extensive geographical spread.^9^

**Research in context**

**Evidence before this study**

Due to the genetic instability of Sabin type 2 poliovirus vaccine and waning population immunity following the global switch from trivalent oral poliovirus vaccine to bivalent oral poliovirus vaccine, an increasing number of new outbreaks caused by circulating type 2 vaccine-derived poliovirus (cVDPV2) have been seeded through the use of monovalent oral poliovirus vaccine type 2 (mOPV2). This increase in new cVDPV2 outbreaks accelerated the development of a more genetically stable type 2 oral vaccine: the novel oral poliovirus vaccine type 2 (nOPV2). The nOPV2 has completed phase 1 and 2 clinical trials in adults in Belgium and in infants and children in Panama, and it was found to be safe and comparably immunogenic to mOPV2. An immunogenicity study in Tajikistan conducted during the nOPV2 outbreak response documented similar seroconversion rates to those observed in clinical trials.

**Added value of this study**

We assessed the seroprevalence against type 2 poliovirus after two rounds of vaccination campaigns for cVDPV2 outbreak response in Liberia. The data unexpectedly demonstrated low immunity in children who reported receiving two doses of nOPV2.

**Implications of all the available evidence**

Our data provide the first assessment of nOPV2 performance in outbreak response in the African continent. We believe that the lessons learned from Liberia are applicable to the global polio eradication programme and further assessments of nOPV2 immunogenicity in different settings should be done.

In November, 2020, a novel oral poliovirus vaccine type 2 (nOPV2) received recommendation under the WHO Emergency Use Listing (EUL) procedure for use in response to cVDPV2 outbreaks.^10^ The nOPV2 is a modified version of Sabin oral poliovirus vaccine type 2 with increased genetic stability and therefore a reduced risk of generating cVDPV.^11^ The nOPV2 has completed phase 1 and phase 2 clinical trials, and it was found to be safe and comparably immunogenic to mOPV2.^12^

In response to a cVDPV2 outbreak in Liberia, two campaigns of nOPV2 vaccination were conducted nationwide in March and May, 2021. It was the second country in the world, after Nigeria, to use nOPV2 for cVDPV2 outbreak response. This use of nOPV2 under EUL provides an opportunity to collect data on the effectiveness of the vaccine in the context of an outbreak response. The aim of this study was to measure seroprevalence against serotype 2 poliovirus after two rounds of nOPV2 supplementary immunisation activities in Liberia.

## Methods

### Study setting

Liberia recorded its first cVDPV2 isolation through environmental surveillance in December, 2020, and its first confirmed case of cVDPV2 infection in May, 2021, in Bong County.^5^ These isolates were genetically linked to an outbreak in Côte D’Ivoire, from a cVDPV2 genetic emergence that originated in Nigeria. Liberia declared the outbreak a National Public Health Emergency in February, 2021, and conducted two nationwide supplementary immunisation activities with nOPV2 in March and May, 2021, with the aim to cover all eligible children with two doses, targeting a total of 972 870 children younger than 5 years.

The current polio vaccination schedule in the national immunisation programme consists of four doses of bOPV: one administered at birth and the following at 6, 10, and 14 weeks of age, plus one dose of IPV administered at 14 weeks. IPV has been included in the routine immunisation programme since 2017.^13^ There have been 11 supplementary immunisation campaigns in Liberia with oral poliovirus vaccines between January, 2016, and January, 2020, for children under 5 years: two tOPV and two bOPV in 2016, three bOPV in 2017, two bOPV in 2018, and two bOPV in 2019. There were no bOPV campaigns in 2020 or 2021 because of the COVID-19 outbreak.

### Study design

This was a cross-sectional, community-based, serological survey conducted in July, 2021, approximately 4 weeks after the end of the second round of the nOPV2 vaccination campaign. Children younger than 59 months residing in Liberia at the time of the study were eligible for enrolment. Ethics approval was obtained from the University of Liberia-Pacific Institute for Research & Evaluation Institutional Review Board and WHO Ethical Review Committee.

Clustered sampling methodology was used in four geographical regions of Liberia (northwestern, north central, south central and southeastern); however, sampling in the southeastern region (counties Maryland, Sinoe, Grand Gedeh, Grand Kru, and River Gee) was not possible because of reduced accessibility during the rainy season. Simple random sampling of households within the clusters was used to identify eligible participants with an urban-rural ratio per cluster of 65:35. Staff visited selected households and, if there were eligible children present, one child was randomly selected per household based on a simple random numbering system. Parent or guardian informed consent was required for enrolment. Children acutely or chronically ill at the time of enrolment or requiring hospital admission were excluded from the study.

The geographical demarcation, random identification of community clusters, and random selection of households within the targeted community clusters were done in collaboration with the Ministry of Health's Vital Statistics and Research Units, including the Liberia Institute of Statistics and Geo-Information Services. Experienced data collectors were recruited and trained by the Ministry of Health and study teams about the vaccination campaigns, community engagement and entry strategies, health-system-related data collection procedures, research methods, biological sample collections and processing procedures, cultural and maternal sensitivities, and ethical issues.

A demographic questionnaire including vaccination history was answered by the parent or legal guardian of the child. Sex was self-reported as a classification of male or female based on biological distinction. The vaccination history collected included possession of a vaccination card and vaccination history for poliovirus vaccines. Participation in the nOPV2 vaccination campaign was determined by parental recall as this information is not included in the vaccination cards. A trained phlebotomist collected a dried blood spot (DBS) on Whatman 903 cards (Sigma-Aldrich, St. Louis, MO, USA) using a fingerprick technique. These were packaged and stored at –20°C before shipment.

The DBS cards were shipped to the US Centers for Disease Control and Prevention in Atlanta, GA, USA, and tested for the presence of neutralising poliovirus antibodies for all three poliovirus serotypes using standard microneutralisation assays (PMID 26983734). We defined seropositivity as a reciprocal antibody titre of ≥8 (≥3, in log_2_ scale).^14^

### Outcomes

The primary objective of this study was to measure seroprevalence and titres of antibodies against type 2 poliovirus in children aged 0–59 months living in the areas targeted by two nOPV2 vaccination campaigns. The secondary objective was to measure seroprevalence against poliovirus types 1 and 3 in the same population.

### Statistical analysis

A sample size of 493 (rounded to 500) was calculated for a 95% confidence level with a precision of approximately ±5% based on the assumption of a type 2 seroprevalence of 80% (indicated by nOPV2 clinical trial data), a 30% attrition rate (due to low-quality DBS, non-responsive households, or refusals), and using the WHO recommended intraclass correlation coefficient of 1/64, selecting six children per cluster with 58 clusters.^15^ Seroprevalence is reported as a proportion with Clopper-Pearson 95% binomial CIs. The reciprocal antibody titres are reported on the log_2_ scale as median values with IQR.

Children younger than 6 months were excluded from risk factor analysis (n=61), giving a sample size of 375. This was done to minimise potential bias that may be due to the presence of maternal antibodies in children younger than 6 months. The difference in proportions between two groups were calculated using Fisher's exact test, due to small sample sizes within some groups.

### Role of the funding source

WHO employees participated in the study design, data collection, data analysis, data interpretation, and writing of the report. Rotary International had no role in study design, data collection, data analysis, data interpretation, or writing of the report.

## Results

**Figure 1:**
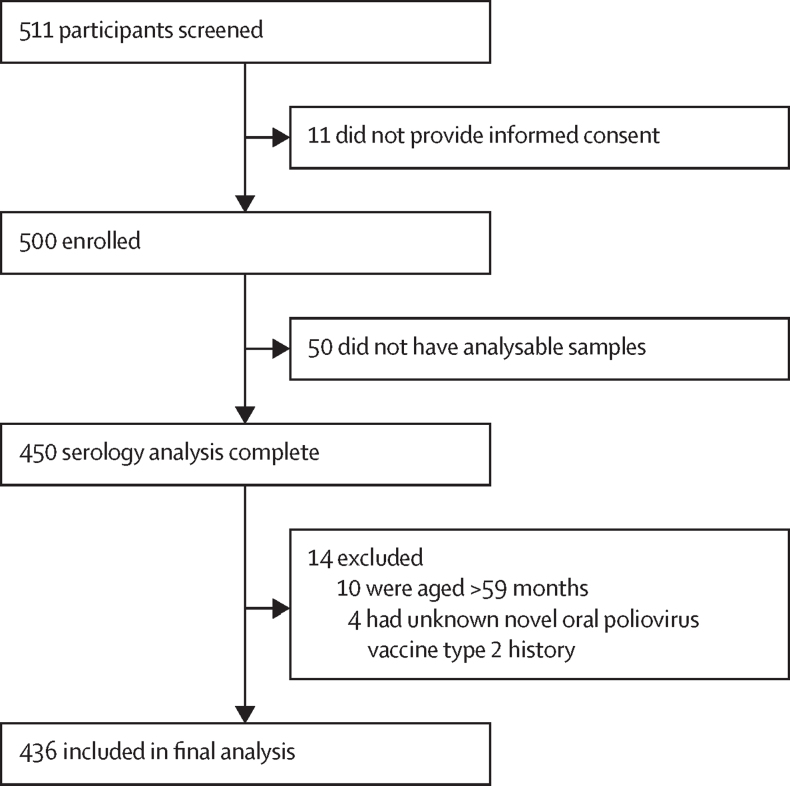
Study profile

Between July 5 and July 11, 2021, 511 individuals were screened of whom 500 (98%) were enrolled in the study (figure 1). 11 (2%) of 511 screened individuals were not enrolled because informed consent was not obtained (for reasons including the parent or guardian was not present, refusal to participate in the study, other engagements or priorities, and lack of availability). Of the 500 enrolled individuals, 64 were subsequently excluded from the final analysis (50 [10%] had their DBS samples misplaced during shipment to the laboratory, ten [2%] were outside the eligible age range, and four [1%] had unknown nOPV2 history). The demographics of excluded individuals are provided in the appendix (p 1). Therefore, a total of 436 (87%) of 500 enrolled individuals were included in the final analysis (figure 1).

The baseline demographic characteristics of the 436 study participants are summarised in table 1. 209 (48%) participants were male, and the average age at the time of sampling was 26 months. A vaccination card was available for 312 (72%) participants. In total, 272 (62%) of all 436 participants were reported via a mixture of vaccination cards and parental recall to have received one dose of IPV, 24 (6%) received no dose of IPV, and 140 (32%) received unknown IPV history. For the 312 with a vaccination card, 263 (84%) were reported to have had one dose of IPV, 19 (6%) had no dose of IPV, and 30 (10%) had unknown IPV history. In total, over the two nOPV2 campaigns, 371 (85%) of 436 participants were reported via parental recall to have had two nOPV2 doses, 43 (10%) of 436 had one nOPV2 dose, and 22 (5%) of 436 had no nOPV2 doses.

**Table 1:** Baseline demographics and vaccination history of study participants

		**Participants (n=436)**
Sex		
	Male	209 (48%)
	Female	227 (52%)
Mean age, months (range)		26 (0–59)
Age distribution		
	0–5 months	61 (14%)
	6–11 months	48 (11%)
	12–35 months	189 (43%)
	36–59 months	138 (32%)
Location (county)		
	Bomi	48 (11%)
	Bong	43 (10%)
	Grand Bassa	75 (17%)
	Grand Cape Mount	11 (3%)
	Lofa	22 (5%)
	Margibi	25 (6%)
	Monteserrado	162 (37%)
	Nimba	25 (6%)
	Rivercess	25 (6%)
Vaccination card available		312 (72%)
IPV received		
	No	24 (6%)
	Yes	272 (62%)
	Unknown	140 (32%)
Number of bOPV doses received in RI		
	1	35 (8%)
	2	91 (21%)
	3	178 (41%)
	4	36 (8%)
	Unknown	96 (22%)
Median number of bOPV doses received in SIA		3 (2–4)
nOPV2 received in first SIA round		383 (88%)
nOPV2 received in second SIA round		402 (92%)
Total nOPV2 doses received		
	0	22 (5%)
	1	43 (10%)
	2	371 (85%)

Data are n (%), mean (range), or median (IQR). bOPV=bivalent oral poliovirus vaccine. IPV=inactivated poliovirus vaccine. nOPV2=novel oral poliovirus vaccine type 2. RI=routine immunisation. SIA=supplementary immunisation activities.

**Figure 2:**
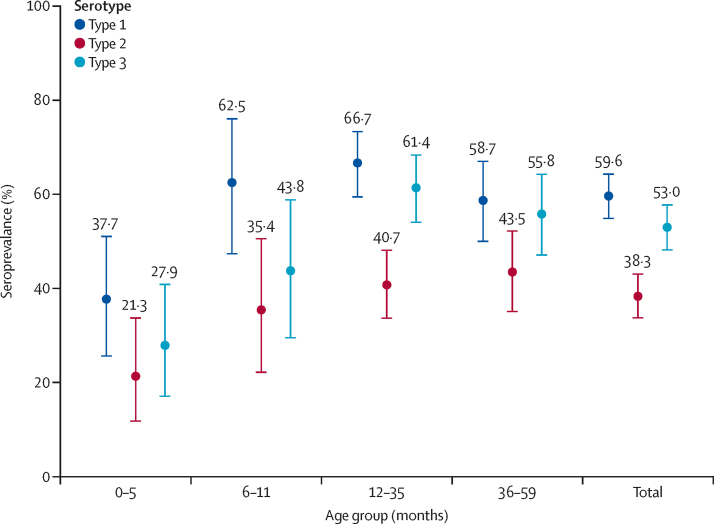
Seroprevalence against poliovirus types 1, 2, and 3 by age group (n=436) Proportion estimate is shown for age groups and all participants (total). Binomial 95% CIs are indicated by vertical lines.

The overall seroprevalence against poliovirus type 2 was 38·3% (95% CI 33·7–43·0; 167 of 436 participants; figure 2). There was evidence of a significant difference in type 2 seroprevalence in children younger than 6 months—21·3% (95% CI 11·9–33·7; 13 of 61)—compared with children aged 6 months or older—41·0% (95% CI 36·0–46·2; 154 of 375; p=0·005). Two doses of nOPV2 were reported for 47·5% (95% CI 34·5–60·7; 29 of 61) of children younger than 6 months, compared with 91·2% (95% CI 87·9–93·9; 342 of 375) in children aged 6 months or older (p<0·0001). The total seroprevalence was 59·6% (95% CI 54·9–64·3; 260 of 436) against poliovirus type 1 and 53·0% (48·2–57·7; 231 of 436) against poliovirus type 3 (figure 2).

The type 2 seroprevalence in children aged 6 months or older (n=375) who were reported to have had no doses of nOPV2 was 37·5% (95% CI 8·5–75·5; three of eight) compared with 28·0% (95% CI 12·1–49·4; seven of 25) who were reported to have had one nOPV2 dose, and 42·1% (36·8–47·5; 144 of 342) who were reported to have had two nOPV2 doses (table 2). There was no evidence of an association between nOPV2 vaccination history and seroprevalence (p=0·39). Individuals aged 6 months or older who were reported to have had one dose of IPV had a type 2 seroprevalence of 43·0% (95% CI 36·6–49·6; 102 of 237) compared with those who were reported to have had no IPV dose (36·4%, 10·9–69·2; four of 11) or those who had unknown history (37·8%, 29·3–46·8; 48 of 127; table 2). There was no evidence of an association between IPV vaccination and type 2 seroprevalence when calculated for all participants (p=0·58) or only those that had vaccination cards (n=260, p=0·90). In addition, we found no evidence of an association between sex (p=0·35), age group (p=0·62), or location (p=0·13; table 2).

**Table 2:** Association between poliovirus type 2 seroprevalence and demographic factors and vaccination history, in individuals aged 6 months and older (n=375)

		**Seroprevalence**		**Fisher's exact p value**
		n/N	% (95% CI)	
Sex		..	..	0·35
	Male	82/188	43·6% (36·4–51·0)	..
	Female	72/187	38·5% (31·5–45·9)	..
Age		..	..	0·62
	6–11 months	17/48	35·4% (22·2–50·5)	..
	12–36 months	77/189	40·7% (33·7–48·1)	..
	36–59 months	60/138	43·5% (35·1–52·2)	..
Number of nOPV2 doses reported		..	..	0·39
	0	3/8	37·5% (8·5–75·5)	..
	1	7/25	28·0% (12·1–49·4)	..
	2	144/342	42·1% (36·8–47·5)	..
IPV dose reported		..	..	0·58
	No	4/11	36·4% (10·9–69·2)	..
	Yes	102/237	43·0% (36·6–49·6)	..
	Unknown	48/127	37·8% (29·3–46·8)	..
Location (county)		..	..	0·13
	Bomi	9/43	20·9% (10·0–36·0)	..
	Bong	17/40	42·5% (27·0–59·1)	..
	Grand Bassa	33/63	52·4% (39·4–65·1)	..
	Grand Cape Mount	3/7	42·9% (9·9–81·6)	..
	Lofa	6/18	33·3% (13·3–59·0)	..
	Margibi	10/23	43·5% (23·2–65·5)	..
	Montserrado	62/143	43·4% (35·0–51·2)	..
	Nimba	6/19	31·6% (12·6–56·6)	..
	Rivercess	8/19	42·1% (20·3–66·5)	..

IPV=inactivated poliovirus vaccine. nOPV2=novel oral poliovirus vaccine type 2.

**Figure 3:**
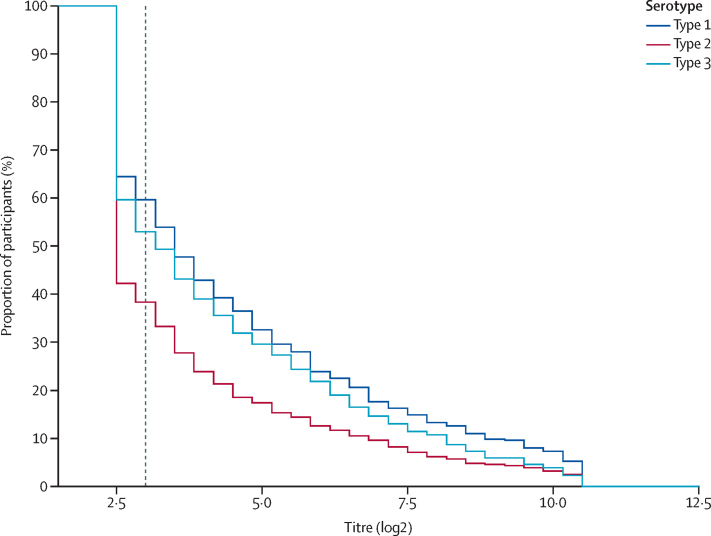
Reverse cumulative curves for the analysis of poliovirus neutralising antibody titres for polio serotypes 1, 2, and 3 Antibody titres on log_2_ scale are shown for serotypes 1, 2, and 3 as a proportion of participants with a neutralising antibody titre above that presented at the intersect. An antibody titre higher than 3 is considered seropositive, indicated by the vertical dashed line.

The median reciprocal antibody titres in log_2_ scale were 3·50 (IQR 2·5–5·83) for type 1, 2·50 (2·50–3·83) for type 2, and 3·17 (2·30–5·50) for type 3 (figure 3).

## Discussion

Our study revealed an unexpectedly low poliovirus type 2 antibody seroprevalence (38%), after two national campaigns with nOPV2 in children younger than 5 years, in a setting where around 60% of children were reported (mainly through vaccination cards, with some via parental recall) to have received IPV in routine immunisation. The median and distribution of type 2 antibody titres were low and correlated with the finding of low seroprevalence. In phase 1 and 2 clinical trials, the immunogenicity of nOPV2 was assessed to be non-inferior to Sabin-based mOPV2,^16^, ^17^, ^18^ and a similar study from Tajikistan reported a seroprevalence above 80% after two doses of nOPV2.^19^ In our study, we found no evidence of an association between type 2 seropositivity in children aged 6 months or older and a reported vaccination history of nOPV2 (p=0·39) or IPV (p=0·58).

Population type 2 immunogenicity in this setting can be attributed to vaccination with nOPV2, IPV administered in routine immunisation, and exposure to asymptomatic infection with circulating virus. One dose of IPV administered at 14 weeks as in the Liberia routine immunisation programme seroconverts approximately 41% of infants,^20^ whereas the prevalence of asymptomatic infection in an endemic area has been estimated at 5%.^21^ It has been documented that immune responses to oral mucosal vaccines in low-income countries are reduced compared with industrialised countries.^22^ A range of factors have been implicated, including the high incidence of enteric infections, malnutrition, diminished vaccine potency, and interference by maternal antibodies. Several studies have demonstrated a reduced immunogenicity of oral poliovirus vaccine in low-income settings, with evidence of an inhibitory effect of concurrent enteric infections on oral poliovirus vaccine response.^23^, ^24^ This reduced immunogenicity of oral poliovirus vaccine might explain the finding of lower-than-expected seroprevalence in our study.

Low nOPV2 campaign coverage despite high reporting from parental recall must be considered. It has been documented that reported vaccination history is imperfect and variable, with parental recall most often biased towards overestimating coverage.^25^

A polio vaccination campaign must aim for immunisation coverage of more than 90% for both rounds with no persistently missed children.^26^ For the two nOPV2 campaign rounds in Liberia, the immunisation coverage was assessed through administrative estimates (a crude estimate calculated by the number of vaccine doses distributed divided by the size of the target population) and lot-quality assurance sampling (LQAS, an independent, randomised method where individuals are randomly selected and checked for vaccination status using clustered sampling).^26^ The administrative estimate was 90% for round one and 103% for round two; however, more robust estimates from LQAS were 33% for round one and 79% for round two. These LQAS estimates are lower than target campaign coverage and parental recall in our study (88% and 92% reported receiving doses in the first and second round, respectively) and, if accurate, could further explain the low seroprevalence.

Poliovirus antibody seroprevalence was 60% against type 1 and 53% against type 3, both achieved through a combination of bOPV and IPV administered in routine immunisation. The joint assessment of immunisation coverage in Liberia by UNICEF and WHO from 2021 estimated coverage of 64% for the third bOPV dose and 64% for IPV.^27^ Seroprevalence surveys with similar study designs have been implemented recently in Cameroon and Chad.^28^, ^29^ In Cameroon in 2020, a higher seroprevalence was documented against poliovirus type 1 (87%) and type 3 (79%), while the estimated coverage for third dose bOPV and IPV were similar to those in our study (70% and 69%, respectively).^28^ Similarly in Chad, in 2019, the seroprevalences against type 1 (91%) and type 3 (83%) were higher than those in our study, with lower third dose bOPV (47%) and IPV (49%) coverage.^29^ However, population immunity is also attributable to supplementary immunisation activities, the frequency of which differs from country to country. Our findings of low seroprevalence against type 1 and 3 in Liberia are not unexpected given the suboptimal coverage of the routine immunisation programme; however, they are substantially lower than found in Cameroon and Chad.

Our study had some limitations. The vaccination history with IPV was not consistently reported, and a large proportion in our sample had unknown or questionable IPV history. This lack of data made it difficult to assess the contribution of IPV to type 2 seroprevalence. As the participation in nOPV2 campaigns is not included on vaccination cards, parental recall was used to determine reported nOPV2 doses, which has limitations in accuracy. Information on temperature monitoring and the cold chain within the country was not available, with heat exposure known to affect vaccine potency. Additionally, degradation of DBS during transport cannot be excluded, as DBS cards were shipped without desiccant or humidity indicators. If DBS cards were exposed to high humidity, the levels of neutralising antibodies against all three serotypes would be negatively affected.

The nOPV2 is a new tool for cVDPV2 outbreak response introduced in the global poliovirus eradication programme. Understanding its immunogenicity in different settings is an important step towards effective use of this vaccine and our findings provide preliminary evidence within the complex assessment of safety and immunogenicity of nOPV2. When planning cVDPV2 outbreak response activities, the global polio eradication programme should take into consideration our findings, which indicate potentially decreased nOPV2 immunogenicity in a setting previously associated with decreased oral poliovirus vaccine immunogenicity.

## Data sharing

Individual participant data will not be made available. The corresponding author can be contacted for inquiries regarding study protocol, the statistical analysis plan, and the informed consent form.

## Declaration of interests

We declare no competing interests.
