# Supplementary material for: Poliovirus antibodies following two rounds of campaigns with a type 2 novel oral poliovirus vaccine in Liberia: a clustered, population-based seroprevalence survey
Source: Lancet Glob Health. 2023 May 16;11(6):e917–23. doi: 10.1016/S2214-109X(23)00116-X (PMC10187988; doi:10.1016/S2214-109X(23)00116-X)
Supplement: Supplementary appendix [file mmc1.pdf]

THE LANCET
Global Health

Supplementary appendix

This appendix formed part of the original submission and has been peer reviewed. We post it as supplied by the authors.

Supplement to: Kennedy SB, Macklin GR, Mason Ross G, et al. Poliovirus antibodies following two rounds of campaigns with a type 2 novel oral poliovirus vaccine in Liberia: a clustered, population-based seroprevalence survey. *Lancet Glob Health* 2023; **11:** e917–23.

Supplementary Materials

Table S1 – Demographics of enrolled participants that were not included in final analysis (n = 64)

**Variable**	**Number (%)**
Total	64
**Sex = Male**	39 (60·9)
**Age in months, mean (range)**	30 (0, 86)
**Age distribution**	
0-5 months	11 (17·2)
6-11 months	6 (9·4)
12-35 months	22 (34·4)
36-59 months	14 (21·9)
60-86 months	11 (17·2)
**Location (County)**	
Bomi	2 (3·1)
Bong	7 (10·9)
Grand Cape Mount	14 (21·9)
Lofa	3 (4·7)
Montserrado	13 (20·3)
Nimba	25 (39·1)
**Vaccination cards available = Yes**	42 (65·6)
**IPV received**	
No	13 (20·3)
Yes	27 (42·2)
Unknown	24 (37·5)
**Number of bOPV doses received in RI**	
1	5 (7·8)
2	9 (14·1)
3	21 (32·8)
4	20 (31·2)
Unknown	9 (14·1)
**Number of bOPV doses received in SIA, median (Q_1 - Q_3)**	6 (5, 7)
**nOPV2 received in 1st SIA round**	
No	7 (10·9)
Yes	55 (85·9)
Unknown	2 (3·1)
**nOPV2 received in 2nd SIA round**	
0	8 (12·5)
1	52 (81·2)
Unknown	4 (6·2)

**Total nOPV2 doses received**	
0	3 (4·7)
1	9 (14·1)
2	48 (75·0)
Unknown	4 (6·2)

Abbreviations: bOPV = bivalent oral poliovirus vaccine; IPV = inactivated poliovirus vaccine; nOPV2 = novel oral poliovirus vaccine type 2; RI = routine immunization; SIA = supplementary immunization activities. $Q_1 =$ lower (25th percentile) quantile; $Q_3 =$ upper (75th percentile) quantile.
